# Use of Heating Methods and Xylose to Increase Rumen Undegradable Protein of Alternative Protein Sources: 1) Peanut Meal

**DOI:** 10.3390/ani13010023

**Published:** 2022-12-21

**Authors:** Fernanda Rigon, David A. B. Pereira, Kalista E. Loregian, Elaine Magnani, Marcos I. Marcondes, Renata H. Branco, Pedro D. B. Benedeti, Eduardo M. Paula

**Affiliations:** 1 Centro APTA Bovinos de Corte, Instituto de Zootecnia, Sertãozinho 14160-970, SP, Brazil; 2 Department of Animal Science, Universidade do Estado de Santa Catarina, Chapecó 89815-630, SC, Brazil; 3 Department of Animal Sciences, Washington State University, Pullman, WA 99164, USA

**Keywords:** beef cattle, ruminal degradation, protein degradation, protein feedstuff

## Abstract

**Simple Summary:**

Protein is a nutrient with a high cost in beef production. Thus, it is crucial to study techniques that improve animal protein utilization. Furthermore, the more productive the animal, the higher its rumen undegradable protein (RUP) requirements. This protein escapes ruminal fermentation to be digested in the intestine. Peanut meal is a feasible byproduct with excellent protein content but low RUP. This problem is commonly solved by submitting the feed to heat processing methods (such as autoclaves or conventional and microwave ovens) with or without treatment with sugars (such as xylose). Thus, here, we submit peanut meal to these different heat techniques, with and without xylose treatment, aiming to increase its RUP content. Overall, our results suggest that the tested methods effectively increased the RUP content of the peanut meal. The best treatments were as follows: for an autoclave, xylose-treated peanut meal with 24 min of heating; for a conventional oven, 60 min of heating for both xylose-treated and -untreated peanut meals; and for a microwave oven, xylose-treated peanut meals with 4 and 6 min of heating. Nevertheless, further research is recommended to evaluate the effects of these ingredients on the parameters of ruminal fermentation and animal performance.

**Abstract:**

Peanut meal has an excellent total protein content but also has low rumen undegradable protein (RUP). High-performance ruminants have high RUP requirements. We aimed to evaluate the effects of processing peanut meal with an autoclave and conventional and microwave ovens, with and without using xylose on its ruminal kinetics degradation parameters and intestinal digestibility (ID). In situ studies were conducted to determine dry matter (DM) and crude protein (CP) rumen degradation kinetics. In vitro studies were conducted to evaluate intestinal digestibility (ID). The control treatment had a greater fraction A for DM and CP than peanut meals processed with an autoclave or conventional oven. The control had greater kd for CP compared with the microwave. The addition of xylose decreased fraction A, the degradation rate of fraction B (kd) and RUP, and increased the protein B fraction of autoclaved peanut meal. We observed a decrease in effective degradability (ED) and increased RUP for processed treatments in all experiments compared with the control. Processing methods did not affect the protein ID of autoclaved peanut meal compared to the control. An interaction between xylose and heating time was observed, where increasing heating time linearly reduced the ID of xylose-untreated treatments. Overall, these results suggest that the tested methods effectively increased the RUP content of peanut meal.

## 1. Introduction

High-performance animals fed with high-concentrate diets in the feedlot typically have high carcass weight gain, which increases beef cattle productivity [1,2]. Furthermore, high-performance animals require more rumen undegradable protein (RUP) to reach metabolizable protein requirements [3,4]. As such, the protection of dietetic protein from ruminal fermentation is essential to increase amino acid utilization, decrease nitrogen waste, and improve feed efficiency [5,6]. Moreover, the increase in the RUP of feedstuffs (and/or diet) may reduce greenhouse emissions from compounds released in the AA fermentation process [7]. Thus, using sufficient RUP ingredients may allow high-producing animals to express their maximum potential [8]. Therefore, there is a demand to maximize animals’ nutrient utilization from feed to improve the profitability of beef cattle production systems [9].

The ruminal degradation of protein sources may vary according to their natural characteristics or with the use of different processing methods. Heating with or without the addition of sugar (xylose) is a method that has shown the potential to increase RUP [10]. Treating a feed with heat may cause strong binding among proteins and carbohydrates [11,12]. This bond is a protection that decreases ruminal protein fermentation, but it is reversible due to the low pH of the abomasum [13]. Furthermore, treatments with reducing sugars such as xylose might further strengthen this bond [14]. Thus, these methods are interesting from an environmental perspective since no chemical ingredients are used in the process. However, an excess of heat exposure (time or intensity) might also protect the protein from post-rumen compartment digestion [15,16]. Studies have reported the effects of autoclaving [17], toasting [18], microwave heating [19,20], and xylose treatment [21] on the improvement of the RUP content of protein sources. However, there are still contradictory results regarding these methods [5,22]. Furthermore, there is no definition of a correct process method that maximizes protein use for other feeds (such as peanut meal) since most studies mainly have evaluated soybean meal.

Soybean meal is the most used protein source in Brazilian feedlot systems for beef cattle [23]. However, due to its high cost, nutritionists have been seeking an alternative feed to improve profitability [23]. Peanut meal is an alternative protein source that might replace soybean meal in ruminant diets [23,24]. However, this feedstuff has low levels of RUP (18.0% RUP/CP) [25,26]. Thus, peanut meal could be a viable strategy to replace soybean meal if the proportion of RUP could be increased. To the best of our knowledge, the consequences of applying processing methods using heating with or without sugar additives on the ruminal degradation parameters of peanut meal are unknown. Therefore, this study evaluated the effects of processing peanut meal with an autoclave and conventional and microwave ovens, with and without xylose, on ruminal kinetics degradation parameters and intestinal digestibility (ID). We hypothesized that processing methods would protect the peanut meal protein fraction from ruminal degradation and increase RUP without affecting ID, thus improving the nutritional value of peanut meal. There is a companion paper that evaluates the same processing methods with respect to increasing RUP in cottonseed meal [27].

## 2. Materials and Methods

### 2.1. Location, Heating Processing Methods, and Chemical Analysis

This is one of two companion papers that evaluated heating processes with or without xylose to increase the RUP of different protein sources (cottonseed and peanut meal). All experiments were conducted at the Instituto de Zootecnia, Beef Cattle Research Center, Sertãozinho, São Paulo, Brazil. The peanut meal used in the study was obtained via oil extraction by mechanically pressing the peanut kernel, followed by organic solvent (hexane) treatment. Three experiments were performed for the processing method evaluation of peanut meal: autoclave (Exp.1), conventional oven (Exp.2), and microwave oven (Exp.3). The design was the same for the three experiments. Seven treatments were investigated within each experiment: control (feed without xylose and heat processing) and three heating times applied to both xylose-treated (inclusion of 20 g/kg DM; D-(+)-xylose, Sigma Aldrich, Darmstadt, Germany) and -untreated peanut meals. The heating times were 8, 16, and 24 min for the autoclave (127 °C and 117 kpa of pressure); 30, 60, and 90 min for the conventional oven (150 °C); and 2, 4, and 6 min for microwave oven (1000 w, full power). 

All ingredients used in these studies were ground through a 2 mm screen (Wiley mill; Thomson Scientific Inc.) to perform all incubations and analyses. Samples were analyzed for dry matter (DM; method G-003/1), ash (method M-001/1), crude protein (method N-001/1), and ether extract (EE; method G-005/1) according to [28]. The organic matter (OM) was calculated as the difference between the DM and ash contents. For neutral detergent fiber (NDF), samples were treated with alpha thermo-stable amylase omitting sodium sulfite [29] and adapted for the Ankom200 Fiber Analyzer (Ankom Technology, Macedon, NY). The chemical compositions of the experimental ingredients are presented in Table 1, Table 2 and Table 3 for Exp. 1 (autoclave), Exp. 2 (conventional oven), and Exp. 3 (microwave oven), respectively.

### 2.2. In Situ Procedures and Calculations

Three cannulated Nellore steers (Average BW of 397 ± 51 kg) were used for in situ evaluation. Animals were housed in an enclosed barn, restrained in individual pens, and fed a 60:40 forage-to-concentrate diet (60% corn silage, 25% dry ground corn, 13% soybean meal, 0.2% urea, and 1.9% mineral mixture). Steers were adapted to this diet 14 d before the study. During this time, they had free access to feed and water. The feed ingredients were individually weighed in nylon bags (Ankom R510; 50 μm porosity, 400 cm^2^ surface area) and incubated in each animal. The bag surface area-to-mass ratio was 15 mg/cm^2^. For each ingredient, simultaneously in each steer, bags were incubated in the rumen for 2, 4, 8, 12, 24, and 48 h. Filter bags were incubated for each treatment in triplicate in each animal and timepoint, totaling 126 bags/per animal. Filter bags with samples were inserted into a washing laundry bag and then were incubated in the rumen (a lead weight was also added to allow for continual immersion within ruminal contents). Bags were placed into the rumen in the reverse order of incubation hours so that all bags were removed simultaneously for washing. 

Once removed, bags were submerged for 15 min in saline solution with ice to stop microbial activity and detach bacteria from the feed fraction. Then, the bags were washed in a washing machine with running cold tap water until the water was clear. The 0 h bags were not incubated in the rumen but were rinsed in running water with the incubated bags. Bags were then oven-dried at 55 °C for 72 h. After drying, the bags were individually weighed. Residues of each treatment were removed from the bags and placed in a labeled plastic bag to obtain a sample of each treatment per animal/incubation time. Residuals of different timepoints were used to estimate the parameters of ruminal degradation.

The DM and CP degradation profiles were estimated using the Ørskov and McDonald (1979) [30] asymptotic function:

Yt = A + B × (1 − e^−(kdt)^), (1)

where Yt is the fraction degraded in time ‘t’, g/kg; A is the water-soluble fraction, g/kg; B is the potentially degradable water-insoluble fraction, g/kg; kd is the degradation rate of fraction b, h^−1^; and t is time, h.

The effective degradability (ED, g/kg) of DM was calculated using the Denham et al. (1989) [31] model:

ED = A + [B × kd/(kd + kp) × e^-kpt^], (2)

where A is the water-soluble fraction, g/kg; B is the potentially degradable water-insoluble fraction, g/kg; kd is the degradation rate of fraction b, h^−1^; t is time, h; and kp is the rumen passage rate (k) of 0.074 h^−1^, obtained from the equation developed by NRC (2001) [32] for concentrates.

The RUP was calculated as follows:

RUP = B × [kp/(kp + kd)],(3)

where B is the potential degradable water-insoluble fraction, g/kg; kd is the degradation rate, h^−1^; and kp = passage rate, h^−1^. 

### 2.3. Intestinal Digestibility Procedures

For in vitro trials, a system with four 4 L digestion vessels (TE-150, Tecnal Equipamentos Científicos, Piracicaba, SP, Brasil) equipped with a slow rotation and temperature controller was used in a 24 h fermentation batch. The three-step in vitro procedure proposed by Calsamiglia and Stern (1995) [33] and modified by Gargalo et al. (2006) [34] was used to determine the intestinal digestion of RUP. Briefly, 1000 mg (DM basis) from the timepoint of 12 h of in situ incubation for each ingredient was weighed in duplicate in R510 bags (Ankom Technology, Macedon, NY, USA). Next, bags were sequentially incubated with constant rotation at 39 °C with pepsin solution (P-7000, Sigma, St. Louis, MO, USA) for 1 h and with pancreatin solution (P-7545, Sigma) for 24 h. After incubation, bags were rinsed with tap water until effluent water remained clear. Then, samples were oven-dried at 60 °C for 48 h. Finally, the residues in the bags were used to determine the DM and N content.

### 2.4. Statistical Analysis

The DM and CP fractions, ED, RUP, and intestinal digestibility were first determined for each replication and compared using a completely randomized model design. Six contrasts tested differences across the treatments:

- Control versus processing treatments;
- Effect of xylose;
- Linear effect of heating time;
- Quadratic effect of heating time;
- Interaction between xylose and heating time.

All analyses were run separately for each processing method because heating times were different depending on the evaluated processing method used. All analyses were run using the PROC GLIMMIX of SAS (SAS on Demand, online version). Significance was declared when *p* < 0.05, and trends were declared when *p* < 0.10, as the critical probability level for a type I error.

## 3. Results

### 3.1. Autoclave

**Figure 1:**
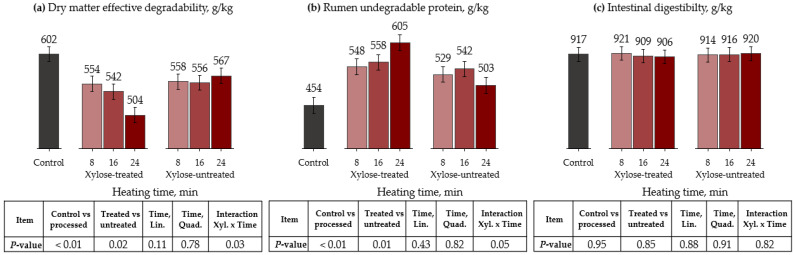
Effects of autoclave heating and xylose treatment on dry matter effective degradability ((**a**), ED), rumen undegradable protein ((**b**), RUP), and intestinal digestibility (**c**) of peanut meal. ED = A + [B × kd/(kd + kp) × e^−kt^] [31]; RUP = B × [kp/(kp + kd)], where A is the water-soluble fraction, g/kg; B is the potentially degradable water-insoluble fraction, g/kg; kd is the degradation rate of fraction b, h^−1^; t is time, h; and kp is the rumen passage rate (k) of 0.074 h^−1^ [32].

The effects of autoclave and xylose inclusion on rumen degradation parameters and the protein ID of peanut meal are presented in Table 4 and Figure 1. For DM kinetics, the control had a greater fraction A and ED than the remaining treatments (*p* < 0.01). There was a tendency for interaction between xylose and time (*p* = 0.08) for fraction B, which responded quadratically—negatively for xylose-treated and positively for xylose-untreated peanut meals. Treatments without xylose had greater kd (*p* = 0.05) and ED (*p* = 0.02) than xylose-treated treatments. There was an interaction between the xylose and processing time for fraction A (*p* = 0.03) and DE (*p* = 0.03). Thus, the autoclave heating time linearly decreased fraction A and DE only in xylose-treated samples. 

Regarding protein kinetics, the control had a greater fraction A and lower RUP than processed treatments (*p* < 0.01). Xylose-untreated samples had a greater fraction A than xylose-treated samples (*p* = 0.01). Furthermore, treatments without xylose had a lower fraction B than those with xylose. We observed a tendency toward greater kd for the xylose-treated group for than the xylose-untreated group (*p* = 0.08). The control had lower RUP than the processed treatments (*p* < 0.01). Moreover, an interaction showed that RUP increased as the processing time increased only for treatments with xylose (*p* = 0.05). Compared with the control, the RUP proportion increased by 21, 23, and 33% when 8, 16, and 24 min of autoclaving were applied to the xylose-treated peanut meal, respectively. Processing methods did not affect protein ID compared to the control (*p* > 0.82).

### 3.2. Conventional Oven

**Figure 2:**
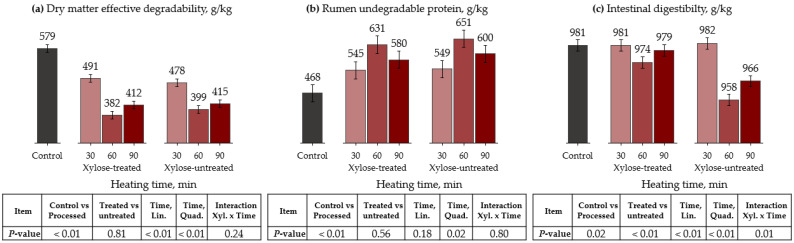
Effects of conventional oven heating and xylose treatment on dry matter effective degradability ((**a**), ED), rumen undegradable protein ((**b**), RUP), and intestinal digestibility (**c**) of peanut meal. ED = A + [B × kd/(kd + kp) × e^−kt^] [31]; RUP = B × [kp/(kp + kd)], where A is the water-soluble fraction, g/kg; B is the potentially degradable water-insoluble fraction, g/kg; kd is the degradation rate of fraction B, h^−1^; t is time, h; and kp is the rumen passage rate (k) of 0.074 h^−1^ [32].

The ruminal degradation parameters of DM and CP, as well as protein ID, are presented in Table 5 and Figure 2, for the conventional oven treatment. The control treatment had greater DM fraction A and ED than the processed treatments (*p* < 0.01). Moreover, ED responded quadratically as heating time increased (*p* < 0.01). On the other hand, the processing methods did not affect the fraction B and kd of DM (*p* > 0.82). 

For CP kinetics, the control had a greater fraction A than the processed treatments (*p* < 0.01). Likewise, processing methods did not affect the fraction B and kd of the protein (*p* > 0.24). The control had lower RUP compared with the processed treatments (*p* < 0.01). Furthermore, heating time had a quadratic effect on RUP, with the highest values reached at 60 min for both xylose-treated and -untreated peanut meals (*p* = 0.02). The processing methods affected ID, reducing it compared with the control (*p* = 0.02). Moreover, there was an interaction between xylose and heating time, where an increased heating time linearly reduced the ID only for xylose-untreated peanut meal (*p* = 0.01).

### 3.3. Microwave Oven

**Figure 3:**
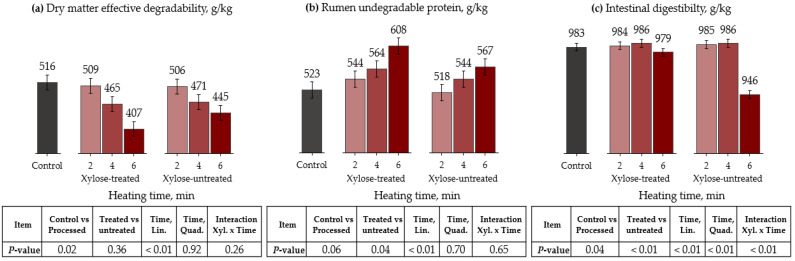
Effects of microwave oven heating and xylose treatment on dry matter effective degradability ((**a**), ED), rumen undegradable protein ((**b**), RUP), and intestinal digestibility (**c**) of peanut meal. ED = A + [B × kd/(kd + kp) × e^−kt^] [31]; RUP = B × [kp/(kp + kd)], where A is the water-soluble fraction, g/kg; B is the potentially degradable water-insoluble fraction, g/kg; kd is the degradation rate of fraction b, h^−1^; t is time, h; and kp is the rumen passage rate (k) of 0.074 h^−1^ [32].

Regarding DM kinetics, the time of microwave heating linearly reduced the fraction A and ED for both xylose-treated and -untreated peanut meals (*p* < 0.01, Table 6 and Figure 3). Furthermore, ED was greater for the control than the processed treatments (*p* = 0.02). However, processing methods did not affect fraction B and kd (*p* > 0.14). 

For CP kinetics, fractions A and B did not differ among treatments (*p* > 0.12). On the other hand, protein kd was greater (*p* = 0.03) and RUP was lower (trend, *p* = 0.06) for the control than for other treatments. Xylose-treated samples had lower kd (trend, *p* = 0.07) and greater RUP (*p* = 0.04) than xylose-untreated peanut meal. Moreover, heating times linearly decreased kd (trend, *p* = 0.06) and increased RUP (*p* < 0.01) for both xylose-treated and -untreated peanut meals. Therefore, the RUP proportion increased by 2, 4, and 9% compared with the control when 2, 4, and 6 min of heating were applied to xylose-treated peanut meal, respectively. The ID was greater for the control than for the remaining treatments (*p* = 0.04). Moreover, there was an interaction between the xylose treatments and heating time, where increasing the heating time linearly reduced the ID for the xylose-untreated peanut meal (*p* < 0.01).

## 4. Discussion

The RUP requirements of beef cattle typically increase as the animal is more productive [3,4]. Thus, processing methods that increase the RUP of common protein sources may improve the efficiency and profitability of high-performance beef cattle animals. Furthermore, heating and xylose treatments have shown the potential to protect the protein from ruminal fermentation [10]. In the present study, we hypothesized that applying different forms and times of heating may change the DM and CP ruminal kinetics of peanut meal. These changes were likely due to alterations in protein structure and chemical profiles, especially in the α-helix-to-β-sheet ratio [35,36]. Alterations in protein structure partly affect the access of enzymes to the gastrointestinal tract, which changes nutrient availability [35]. The α-helix-to-β-sheet ratio is essential since it has a positive correlation with the proportion of protein absorbed in the intestine and a negative correlation with degraded protein balance [35,36]. Indeed, the control treatment had a greater soluble fraction for both DM and CP than processed peanut meals for the autoclave and conventional oven. Heat processing methods can form stable polymers among carbohydrates and proteins that escape ruminal degradation [11,12]. These polymers, in general, are resistant to enzymatic attacks from ruminal microorganisms [37,38]. This type of processing especially alters fraction A, which reduces the availability of this fraction for microbial degradation in the rumen and the extent of ruminal fermentation [39,40]. Our results also suggest that a significant part of the protected DM was indeed the protein fraction since we observed a reduction in fraction A for both DM and CP. 

On the other hand, protein fraction A of the protected feeds did not differ from the control when the treatment microwave oven was applied. Heating the feeds for up to 6 min in the microwave was likely not enough to change fraction A. Others have also observed that the changes promoted by microwave heating have a lesser impact than the heat promoted by conventional ovens [20]. Nevertheless, in our study, the control had greater kd for CP than the microwave oven treatments. Thus, despite the lack of effects for fraction A, microwave oven heating was enough to decrease the percentage of protein degraded in the rumen over time compared with the control. Different than protein fraction A, the fraction A of DM decreased with the increasing heating time in the microwave oven, which can be related to the protection of other nutrients, such as carbohydrates.

Xylose is a reducing sugar that may also contribute to heating to decrease ruminal degradation, catalyzing the reaction between the aldehyde groups of xylose and amino acids [21]. Thus, we hypothesized that treating peanut meal with xylose could protect the protein fraction from ruminal degradation. As expected, adding xylose decreased the soluble fraction and kd (trend) and increased the insoluble fraction of the protein of autoclaved peanut meal. These results lead us to believe that xyloses may affect protein solubility due to their binding with amino acids. Thus, the changes in ruminal fermentation impacted fermentation kinetics given that the ED was lower and the RUP was greater for xylose-treated peanut meal. 

Moreover, the linear increase in DM fraction A and the decrease in the ED of DM only for xylose-treated peanut meal indicates that this sugar may potentialize the heating effects on ruminal kinetics. It is also interesting to observe the tendency toward a linear increase in fraction B (For DM and CP) for xylose-untreated peanut meal with increasing heating times, which may indicate that without adding xylose more heating time is needed to change the kinetic parameters of fermentation. On the other hand, the same results were not observed for other processing methods. There was only a tendency toward CP kd reduction in xylose-treated peanut meal for the microwave oven. Moreover, adding xylose did not change the kinetic parameters of the conventional oven processing method. Therefore, this processing method’s longest heating time probably compensates for the xylose’s contribution to protein protection. 

The findings herein confirmed our hypothesis that heating and xylose treatment can protect peanut meal protein from ruminal fermentation. Compared with the control, we observed a decrease in ED and an increase in RUP for all processing methods. These results agree with those that also observed changes in the kinetic parameters of ruminal fermentation [37]. Thus, processing methods could decrease soluble components and consequently reduce the percentage of protein degraded over time in rumen, which is reflected directly in RUP values. Furthermore, heating time also influenced these parameters in conventional and microwave oven treatments. 

Regarding conventional ovens, heating in a conventional oven at 60 min resulted in a significant quadratic effect in lowering ED and increasing RUP. Concerning the microwave oven, we expected a reduction in these parameters since microwaves could potentially change the terminal molecular structure of proteins [41]. Indeed, our results indicated more efficient protein protection from rumen fermentation as the heating time increased. Thus, the effects of longer microwave heating times on RUP and ID should be evaluated in further research. Xylose treatment effects on RUP also follow results for the other kinetic parameters, having greater effects during the autoclave processing method. In addition to the greater RUP for xylose-treated peanut meal, there was an association between sugar and time, resulting in more protein protection as heating time increased. The same results were also observed for the RUP of peanut meal when the microwave technique was used. Therefore, we can conclude that heating in combination with a xylose treatment may increase the RUP of peanut meal. Nevertheless, we have analyzed the ID of these ingredients to ensure that their protein can be digested and absorbed in the intestine. 

Amino acids and peptides can be unavailable at the intestinal level when overheating occurs [37,42]. Thus, the main goal of this study was to obtain heating processing techniques that could increase the RUP of peanut meal without impairing its ID. Regarding the autoclave method, the lack of differences in ID among the treatments suggests that the bond between carbohydrates and proteins could be completely disrupted in post-rumen compartments. Furthermore, the results observed for xylose-untreated peanut meals processed in conventional and microwave ovens indicate that Maillard’s reaction was irreversible for the more significant portion of protein after a particular heating time (30 min for a conventional oven and 4 min for a microwave). Interestingly, these effects were less intense on xylose-treated peanut meals, which suggests that this sugar, bound with protein, is easier to break down in the small intestine.

Our results lead us to conclude that heating plus the addition of xylose may protect protein from ruminal degradation and increase the RUP. The differences in heating time depend on the processing method applied and are likely related to the different ways that each technique applies heat. For example, feedstuff heated in conventional ovens goes from the surface to the interior, resulting in an uneven heat effect on the protein’s structure [43]. Thus, this method requires more processing time to increase RUP; feed heating in an autoclave might be more uniformly distributed throughout the sample because this technique adds moisture and pressure to the process [36]; feedstuff heating in a microwave is faster since the process results in heat throughout the samples [44]. In summary, our results suggest potential treatments for each experiment (heating process). Regarding autoclaves, we highlight the xylose-treated peanut meal with 24 min of heating, which resulted in 32% more RUP digested in the intestine than non-processed peanut meal. For the conventional oven, the 60 min of heating for both xylose-treated and -untreated peanut meals were the most promising treatments. They increased the intestine-digested RUP by 34 and 36%, respectively, compared with the control. Concerning microwave ovens, the best treatments used were the xylose-treated peanut meals with 4 and 6 min of heating, which increased the intestine digestible RUP up to 8 and 16%, respectively. The next step is to run studies to evaluate the rumen fermentation parameters of these ingredients.

## 5. Conclusions

Our results suggest that heating processes using an autoclave plus xylose increased the RUP content of peanut meal without compromising RUP intestinal digestibility. Heating processes via a conventional oven increased the RUP content of peanut meal compared with the control. However, there were no benefits in adding xylose, and longer heating times without xylose decreased intestinal digestibility. Furthermore, heating processes via a microwave oven plus xylose also increased the RUP content without compromising RUP intestinal digestibility; however, intestinal digestibility was compromised when 6 min of heating was applied. Thus, the best results in these experimental conditions were as follows: for the autoclave, xylose-treated peanut meal with 24 min of heating; for the conventional oven, 60 min of heating for both xylose-treated and -untreated peanut meals; and for the microwave oven, xylose-treated peanut meals with 4 and 6 min of heating. Nevertheless, further research is recommended to evaluate the effects of these ingredients on ruminal fermentation parameters and animal performance.

## Figures and Tables

**Table 1:** Chemical composition of experimental ingredients for Experiment 1—Autoclave.

Item	Control	Xylose-Treated			Xylose-Untreated		
		8	16	24	8	16	24
Dry matter, g/kg	910	908	911	910	916	912	914
Organic matter, g/kg DM	946	948	951	947	946	949	947
Crude protein, g/kg DM	628	645	639	643	647	655	650
Neutral detergent fiber, g/kg DM	164	285	300	315	259	254	260

**Table 2:** Chemical composition of experimental ingredients for Experiment 2—Conventional Oven.

Item	Control	Xylose-Treated			Xylose-Untreated		
		30	60	90	30	60	90
Dry matter, g/kg	910	904	936	930	926	921	920
Organic matter, g/kg DM	946	970	948	947	947	946	946
Crude protein, g/kg DM	628	645	639	643	647	655	650
Neutral detergent fiber, g/kg DM	164	310	456	449	263	455	368

**Table 3:** Chemical composition of experimental ingredients for Experiment 3—Microwave Oven.

Item	Control	Xylose-Treated			Xylose-Untreated		
		2	4	6	2	4	6
Dry matter, g/kg	910	919	929	928	918	921	931
Organic matter, g/kg DM	946	946	948	947	945	933	945
Crude protein, g/kg DM	628	610	617	639	630	639	638
Neutral detergent fiber, g/kg DM	164	264	322	240	249	268	278

**Table 4:** Effects of autoclave and xylose inclusion on rumen degradation parameters of peanut meal.

Item ^1^	Control	Xylose-Treated ^2^			Xylose-Untreated ^2^			SEM	*p*-Value				
		8	16	24	8	16	24		Control × Processed	Xyl.-Treated × -Untreated	Time, Lin.	Time, Quad.	Interaction Xyl. × Time
**Dry matter**													
A, g/kg	355	300	299	258	287	286	308	12.5	<0.01	0.47	0.41	0.68	0.03
B, g/kg	582	642	586	607	560	610	578	27.6	0.61	0.22	0.76	0.95	0.08
kd, h^−1^	0.054	0.048	0.053	0.050	0.069	0.058	0.061	0.007	0.75	0.05	0.62	0.80	0.30
**Crude protein**													
A, g/kg	299	158	170	119	191	177	184	15.1	<0.01	0.01	0.15	0.46	0.41
B, g/kg	717	870	807	784	660	770	767	48.4	0.28	0.04	0.83	0.68	0.07
kd, h^−1^	0.038	0.037	0.040	0.041	0.056	0.044	0.051	0.007	0.40	0.08	0.95	0.49	0.30

^1^ A, water-soluble fraction; B, potentially degradable water-insoluble fraction; kd, degradation rate of fraction B; SEM, standard error of the mean. ^2^ Autoclave heating times: 8, 16, and 24 (127 °C and 117 kpa of pressure).

**Table 5:** Effects of conventional oven heating and xylose inclusion on the rumen degradation parameters of peanut meal.

Item ^1^	Control	Xylose-Treated ^2^			Xylose-Untreated ^2^			SEM	*p*-Value				
		30	60	90	30	60	90		Control × Processed	Xyl.-Treated × -Untreated	Time, Lin.	Time, Quad.	Interaction Xyl. × Time
**Dry matter**													
A, g/kg	358	262	247	279	255	225	267	23.2	<0.01	0.49	0.55	0.19	0.78
B, g/kg	653	743	528	466	716	648	756	140	0.95	0.30	0.43	0.52	0.62
kd, h^−1^	0.039	0.033	0.025	0.036	0.034	0.026	0.018	0.007	0.19	0.40	0.36	0.43	0.98
**Crude protein**													
A, g/kg	345	242	235	257	244	244	245	18.7	<0.01	0.97	0.70	0.67	0.86
B, g/kg	871	937	447	501	748	498	662	189	0.28	0.96	0.21	0.19	0.55
kd, h^−1^	0.020	0.022	0.034	0.065	0.028	0.019	0.029	0.017	0.50	0.31	0.24	0.54	0.53

^1^ A, water-soluble fraction; B, potentially degradable water-insoluble fraction; kd, degradation rate of fraction B; SEM, standard error of the mean. ^2^ Heating times: 30, 60, and 90 min in a conventional oven (150 °C).

**Table 6:** Effects of microwave oven heating and xylose inclusion on the rumen degradation parameters of peanut meal.

Item ^1^	Control	Xylose-Treated ^2^			Xylose-Untreated ^2^			SEM	*p*-Value				
		2	4	6	2	4	6		Control × Processed	Xyl.-Treated × -Untreated	Time, Lin.	Time, Quad.	Interaction Xyl. × Time
**Dry matter**													
A, g/kg	247	290	238	202	252	206	204	18.4	0.46	0.15	<0.01	0.38	0.29
B, g/kg	778	811	850	822	816	851	890	37.6	0.14	0.43	0.28	0.65	0.41
kd, h^−1^	0.041	0.028	0.027	0.025	0.035	0.034	0.028	0.007	0.15	0.34	0.47	0.82	0.79
**Crude protein**													
A, g/kg	175	172	168	179	199	155	162	13.5	0.88	0.95	0.27	0.18	0.12
B, g/kg	905	941	964	905	918	923	948	38.7	0.52	0.83	0.93	0.65	0.41
kd, h^−1^	0.038	0.031	0.028	0.022	0.033	0.035	0.029	0.003	0.03	0.07	0.06	0.34	0.43

^1^ A, water-soluble fraction; B, potentially degradable water-insoluble fraction; kd, degradation rate of fraction B; SEM, standard error of the mean. ^2^ Heating times: 2, 4, and 6 min in a microwave oven (1000 w, full power).

## Data Availability

The data presented in this study are available upon request from the corresponding author.
